# Pre-operative templating in THA using a short stem system: precision and accuracy of 2D versus 3D planning method

**DOI:** 10.1186/s10195-022-00634-x

**Published:** 2022-03-22

**Authors:** Patrick Reinbacher, Maria Anna Smolle, Joerg Friesenbichler, Alexander Draschl, Andreas Leithner, Werner Maurer-Ertl

**Affiliations:** Department of Orthopaedics and Trauma, Medical University of Graz, Auenbruggerplatz 5, 8036 Graz, Austria

**Keywords:** Total hip arthroplasty, Templating, Short stem, 2D, 3D

## Abstract

**Background:**

Total hip arthroplasty (THA) is the most successful orthopaedic surgery of the past century. The current study aimed to compare the accuracy of digital planning using 2D versus 3D templating.

**Materials and methods:**

Ninety-five THAs in 90 patients were included in the current study. Pre- and post-operative X-rays (in two planes) and low-dose rotation computed tomography scans from hip to foot were performed. Paired *t*-test and regression analyses were conducted to compare 2D and 3D templating accuracy of the definitive implant.

**Results:**

Cup size planned both with 2D (*p* < 0.0001) and 3D (*p* = 0.012) templating was significantly different from the definitively used cup size. The difference between the 2D-planned and implanted stem size (*p* < 0.0001) was statistically significant. In contrast, there were no significant differences in the 3D-planned and implanted stem size (*p* = 0.181). Three-dimensional templating showed significantly higher accuracy than 2D templating in terms of cup size (1.1 ± 1.4 versus 1.7 ± 1.8; *p* = 0.007) and stem size (0.3 ± 0.6 versus 0.7 ± 0.7; *p* < 0.0001).

With increasing body mass index (BMI), 2D templating of the stem became more inaccurate (*p* = 0.041). Remarkably, 3D templating remained accurate for all components (stem, *p* = 0.533; cup, *p* = 0.479) despite increasing BMI.

**Conclusion:**

Despite extended planning time and increased exposure to radiation, 3D-based planning showed higher accuracy than 2D templating, especially in obese patients. On the basis of our results, we believe that 3D-based pre-operative planning in THA is justifiable and beneficial in patients with increased BMI.

**Level of Evidence:**

III.

## Introduction

Total hip arthroplasty (THA) is known as the most successful orthopaedic procedure of the past century [1]. The operation replaces the damaged hip joint with artificial prosthetic components [2], contributing to pain relief and reduced patient suffering [3]. Over the past decades, improvements in materials, implant design and surgical techniques have increased implant survival [4] and decreased complication rates [5, 6]. Furthermore, patients’ expectations regarding function and longevity have increased because of these improvements [7].

With the increasing number of THAs with uncemented prostheses, the importance of pre-operative templating to restore or correct hip architecture has been extensively debated in the literature [7–10], and more emphasis has been placed on the proper selection of the accurate implant size to avoid complications such as fractures or subsidence [11]. Therefore, accurate pre-operative planning has become an important aspect of determining the correct implant size and position [12–14]. It is regarded as a vital step to successful component implantation [9], increases implant survival [15] and reduces complications associated with surgery [14]. Pre-operative templating is necessary to retain joint biomechanics such as total offset, leg length, centre of rotation and lateralization [16–18]. Femoral offset and leg length are essential parameters that must be restored to improve functional outcomes and long-term survival rates after THA [19–21]. Furthermore, pre-surgical templating is also important from an economic point of view, as a correct estimation of component sizes can avoid the waste of expensive parts.

New technologies, which have made digital templates available, provide innovative techniques that allow more accurate and consistent surgical planning [9, 10, 22–25]. The development of digital pre-operative planning of THA using plain radiographs for 2D [9, 10,12] and computed tomography (CT) for 3D templating [9, 22] improved the accuracy of implant position [7, 9, 22–25]. However, 2D templating still remains the gold standard in clinical practice [26, 27] despite its reported inferior accuracy [7].

This study aimed to compare the accuracy of a 2D and 3D planning tool regarding the size of the implant of an uncemented short stem system (ANA.NOVA Proxy, ImplanTec GmbH, Mödling, Austria) using the MediCAD 2D and 3D software.

## Materials and methods

Ninety consecutive patients who underwent THA between 2016 and 2017 at a single, urban, high-volume university hospital were initially enrolled in the prospective comparative study. Five cases were subsequently excluded owing to incomplete information available, resulting in 90 patients with either unilateral (*n* = 85) or bilateral (*n* = 10) THA at a single institution with a total of 95 implanted THAs eligible for analysis. Eighty-four THAs were performed for primary coxarthrosis (88.4%). In addition, six cases of mild developmental dysplasia of the hip (6.3%), four post-traumatic coxarthroses (4.2%) and one avascular necrosis (1.1%) were also included in the current study.

Outpatient care included functional examination, and imaging was done using plain radiographs. Pre-operatively, X-rays in two planes and a low-dose CT scan were conducted, followed by a post-operative X-ray, 6 weeks, 3 months, 6 months and 1 year after surgery. After that, outpatient care was conducted annually. The pre-operative low-dose CT was done for 3D templating. All the X-rays and CT scans were performed using the same devices with the patient in a standardized (supine) position. Demographic data (age at time of surgery, gender, BMI), duration of surgery, and pre- and post-operative radiographic measurements of hip geometry were collected.

THA was performed in all patients using a new cementless short stem system (ANA.NOVA Proxy, ImplanTec GmbH, Mödling, Austria) and ceramic-on-ceramic head and liners. On the basis of X-ray and CT scans, the sizes of the implant components (i.e. cup and stem) were planned pre-operatively with both software systems (MediCAD 2D and 3D software). All operations were performed by a single consultant surgeon with an anterolateral approach to the hip with the patient in a supine position.

The study procedure followed accepted ethical, scientific and medical standards and was conducted in compliance with recognized international standards, including the principles of the Declaration of Helsinki. Informed consent was obtained from all the participants, and the study protocol was approved by the local ethics committee (28–152 ex 15/16) and a current amendment (received on 21 April 2020).

### Pre-operative digital templating

The MediCAD software system (mediCAD, Hectec GmbH, Altdorf, Germany) was used for pre-operative digital 2D and 3D templating, incorporating essential parameters such as femoral segmentation, acetabulum diameter, hip joint centre, femoral neck axis, femoral stem axis and leg length difference. The pre-operative measurements were carried out independently of the definitively used implants and the surgeon performing surgery.

### Statistical analysis

Statistical analyses were carried out using Stata/SE 15.1 (StataCorp, College Station, TX, USA). Mean values and medians were given with the corresponding standard deviation (SD) or interquartile range (IQR). Differences between 2D- and 3D-planned implants and used implants were compared with a paired *t*-test to calculate the significance. The absolute differences (i.e. positive values only) from 2D- and 3D-planned to definitive implants were calculated for each component (i.e. cup, stem). By reapplying paired *t*-tests, the absolute differences between 2D- and 3D-planned to definitive implants were estimated. Assuming that overlaying soft tissues may impair calibration of the X-rays for the 2D-based pre-operative planning owing to incoherent scattering, the correlation between BMI and the absolute difference of 2D- and 3D-based measurements compared with the definitive implant was estimated with regression analyses. They were performed to assess: (1) any potential association between BMI and Dorr types, (2) the accuracy of 2D- versus 3D-planned implants and (3) any potential improvements in the accuracy of 2D or 3D templating within the period covered by the study. Additionally, a multivariate linear regression analysis was calculated to evaluate 2D versus 3D templating accuracy in relation to the Dorr type. A two-sided *p*-value < 0.05 was considered statistically significant.

**Fig. 1:**
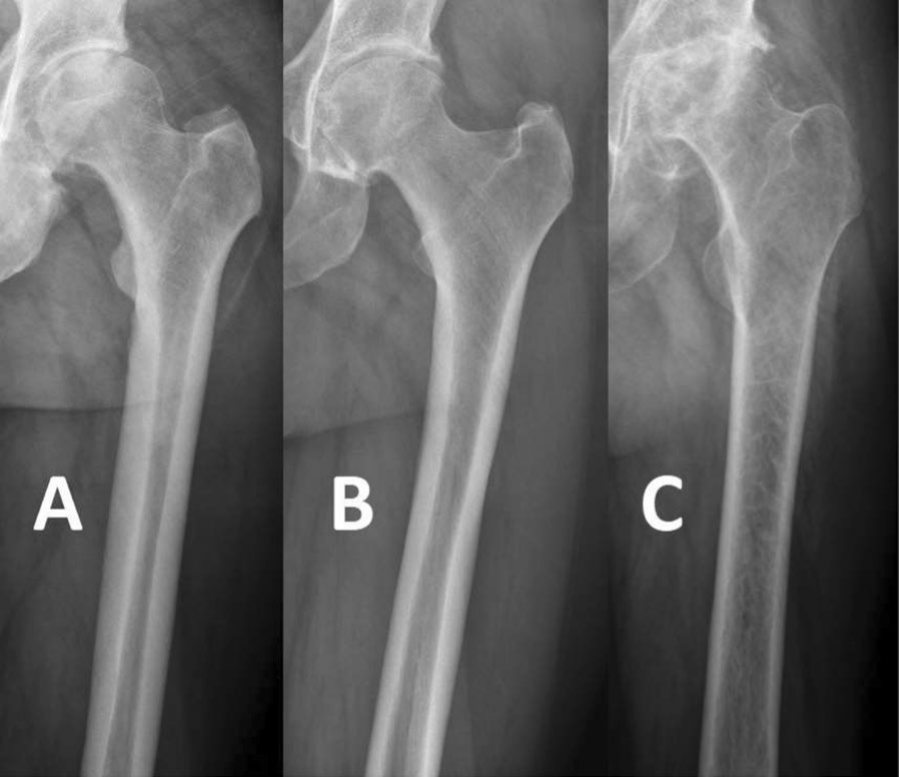
Morphological types described by Dorr

Described by Dorr et al. [28], the three morphological patterns of the femur were named A, B and C (Fig. 1). Dorr types were assessed as follows: type A with a funnel shape and narrow diaphyseal canal; type B exhibits proximal bone loss and widening of the diaphyseal canal; type C with an extensive medullary canal and blurred appearance to the bone cortex due to cortical thinning. The Dorr classification was chosen as it allows quantification of bone quality and anatomy at the proximal femur. Furthermore, depending on Dorr type, the implant itself may be positioned slightly differently and at varying size.

## Results

A single consultant surgeon performed a total of 95 THAs with an anterolateral approach to the hip in a supine patient position independently of the pre-operative measurements conducted by another surgeon who did not perform the surgery. The study included 90 patients (85 unilateral, 10 bilateral) with a mean age of 60.5 years (SD ±7.7 years) at the time of surgery. The study group contained 41 female patients (43.2%). The mean body mass index (BMI) was 28.5 (SD ±4.9). Twenty-four patients had Dorr-type A (25.26%), 59 type B (62.11%) and 12 type C (12.63%). The majority of THAs were performed for primary coxarthrosis (*n* = 84; 88.4%). Rare indications included six cases of mild developmental dysplasia of the hip (6.3%), four post-traumatic coxarthroses (4.2%) and one avascular necrosis (1.1%).

The average pre-operative 2D-based planning time was 5 min compared with 16 min when the 3D-based method was used.

There was a statistically significant difference between planned and implanted cup size both for 2D- (*p* < 0.0001) and 3D-based measurement (*p* = 0.012). While the difference of the implanted stem size in the 2D-planned group (*p* < 0.0001) was statistically significant, there was no significant difference in the 3D-planned implanted stem size (*p* = 0.181; Table 1). In other words, 2D-based planning resulted in a difference of at least two stem sizes in eight cases compared with four cases when 3D-based planning was used.

**Table 1:** Paired *t*-tests for 2D- and 3D-planned implants compared with definitively used implant components

	Implanted	2D		3D	
		Size	*p*-Value	Size	*p*-Value
Cup	54.3 ± 4.0	53.1 ± 3.9	** < 0.0001**	53.8 ± 4.0	**0.012**
Stem	6.1 ± 1.8	5.6 ± 1.8	** < 0.0001**	6.0 ± 1.8	0.181

Significant values are in bold

Regarding the measurement of the planned and implanted cup size, the 3D-based measurement showed significantly higher accuracy than 2D-based measurement when compared with absolute difference (1.1 ± 1.4 versus 1.7 ± 1.8; *p* = 0.007). Furthermore, in terms of measurement and stem size, the 3D-based measurement showed significantly higher accuracy than the 2D-based method (0.3 ± 0.6 versus 0.7 ± 0.7; *p* < 0.0001) (Table 2).

**Table 2:** Paired *t*-test comparing the absolute difference of 2D-based planning and definitively implanted size compared with 3D-based planning of component size and definitive implant

	2D	3D	*p*-Value
	Size	Size	
Cup	1.7 ± 1.8	1.1 ± 1.4	**0.007**
Stem	0.7 ± 0.7	0.3 ± 0.6	** < 0.0001**

Significant values are in bold

**Fig. 2:**
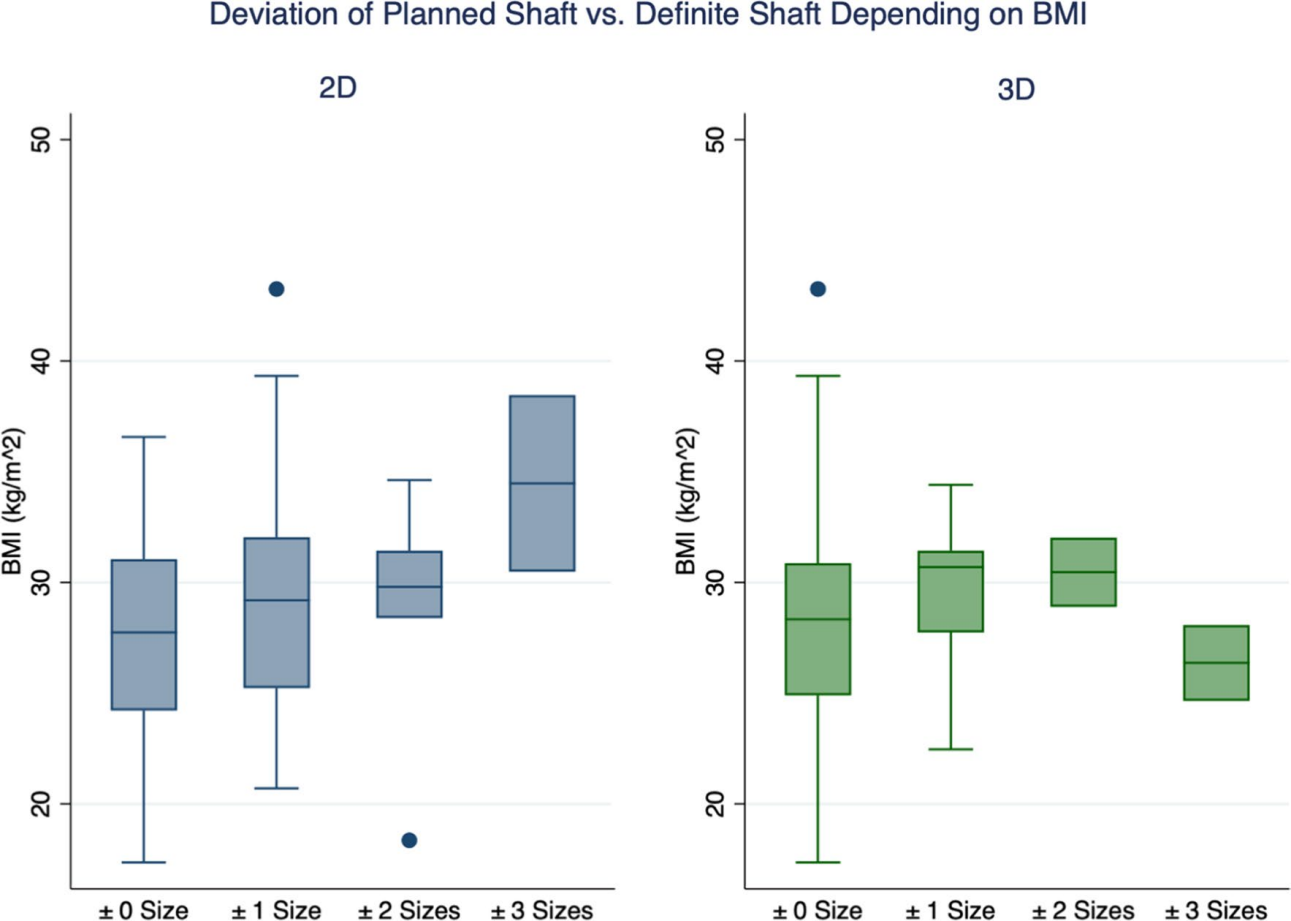
Correlation between accuracy of 2D- and 3D-based measurement of stem size with increasing BMI

With increasing BMI, the 2D-based measurements became more inaccurate regarding stem size (*p* = 0.041). However, at the same time, there was no difference in cup size (*p* = 0.239). Notably, 3D-based measurement remained accurate, despite increasing BMI, for stem size (*p* = 0.533) and cup size (*p* = 0.479; Fig. 2). Moreover, the accuracy of 2D- and 3D-based measurement for stem size did not significantly differ between different Dorr types (*p* = 0.379 and *p* = 0.187, respectively).

Although the accuracy of 2D templating did not improve during the time period covered by the study [*b* = 0.0026 (SE 0.0025); *p* = 0.295], 3D templating showed a tendency to be more accurate when more measurements were performed [*b* = −0.0046 (SE 0.0022), *p* = 0.045].

Remarkably, 3D templating was more accurate in predicting actual stem size compared with 2D templating, irrespective of the Dorr type and BMI (*p* < 0.001; Table 3).

**Table 3:** Multivariate linear regression analysis for the absolute difference from planned to definitively implanted component

Diff*		Coef.	Standard error	*p*-Value	95% CI
Dorr type	*A (ref.)*	1			
	*B*	−0.194	0.112	0.084	[−0.414, 0.026]
	*C*	−0.263	0.164	0.110	[−0.587, 0.061]
BMI (continuous)		0.014	0.010	0.141	[−0.004, 0.033]
Templating	*2D (ref.)*	1			
	*3D*	−0.411	0.093	< 0.001	[−0.594, −0.227]
Constant		0.867	0.339	0.011	[0.198, 1.537]

*Coef.*, coefficient of multivariate linear regression. The coefficient for each of the variables indicate the amount of change one could expect in the different variables (Dorr type A, 8, C, BMI, ...) given a one-unit change in the vlaue of that veriable, given that all other variables in the modelare held constant

*Absolute difference of planned (2D or 3D) implant to definitive implant

## Discussion

We retrospectively analysed the accuracy of 95 pre-operative digital THA templates. According to the present study, 3D templating is more precise in predicting definitively implanted cup and stem size than 2D templating. Furthermore, the former procedure allows reliable prediction of implant size with increasing BMI, while the accuracy of 2D templating decreases steadily.

**Fig. 3:**
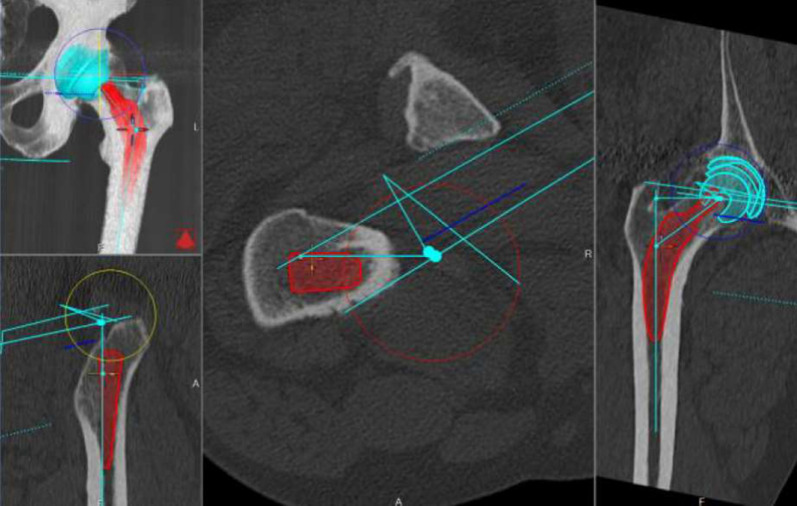
Pictures of 3D templating Hectec mediCAD hip 3D

**Fig. 4:**
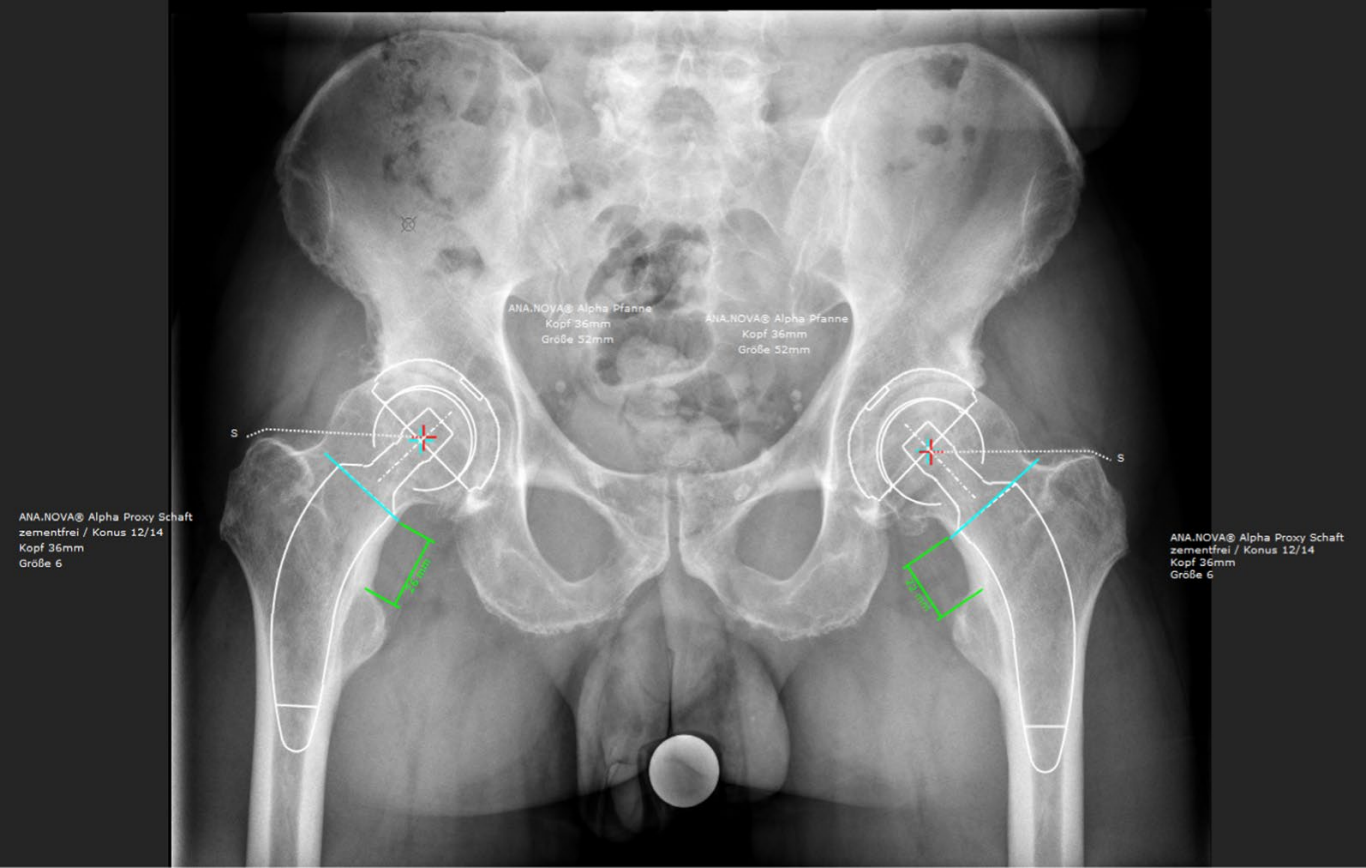
Pictures of 2D templating Hectec mediCAD hip 2D

This study has significant limitations. First, as only one THA system was used, the results are not necessarily applicable to other implant systems, and the results are not convertible to other stems. Second, the majority of THAs in the present study were performed for conventional primary coxarthrosis, as well as a few cases of mild hip dysplasia, post-traumatic arthritis and avascular necrosis. Thus, the accuracy of 2D and 3D templating in more complex anatomical configurations remains unclear. On the other hand, all operations were performed by the same surgeon, allowing unbiased estimation of the reliability of 2D and 3D templating. Furthermore, 3D templating showed an improvement in accuracy over time, possibly due to the physician’s increased experience with the software. Nevertheless, good planning is essential owing to the metaphyseal anchorage of the cementless short stem system and the flat learning curve for implantation (Figs. 3, 4).

Many studies have already shown that CT-based 3D templating is associated with excellent reliability regarding THA component size and alignment [29–31]. In addition, implants to be used for THA in patients with anatomical variances such as developmental dysplasia of the hip or extremely reduced caput–collum–diaphyseal (CCD) angles can be planned more reliably with 3D templating regardless of the surgeon’s experience [29, 31, 32].

Owing to the increasing prevalence of obesity worldwide and its association with comorbidities such as osteoarthritis, more and more patients with a BMI > 30 kg/m^2^ undergo THA [33–35]. However, obese patients are at a significantly higher risk of developing post-operative complications, including re-admission, dislocations, superficial wound healing deficits and periprosthetic joint infections [36]. Therefore, reliable pre-operative templating is necessary to decrease the operation time and, thus, possible complications. Several studies have shown that meticulous preparation reduces operation time and complications in case of increased BMI [34, 37]. However, the increased Compton scattering and photoelectric effect with higher BMI may impair 2D templating. At the same time, CT-based 3D templating on both the axial and sagittal planes may still allow accurate planning of implant components. Despite the increasing BMI, the 3D measurement remained accurate for the stem and the cup, while the 2D templating lost its accuracy.

Furthermore, upon multiple linear regression, 3D templating was more accurate than 2D templating in the context of definitively used stem size, irrespective of BMI or Dorr type.

Although we still lack evidence that a perfect match between planned and actual implant size (and position) has a positive impact on clinical outcome [7], precise pre-operative planning is vital to shorten the surgery time and thus potentially reduce the infection rate [38].

It should be noted that only low-dose CT scans should be performed for pre-operative 3D templating, as the radiation exposure of CT scans is relatively higher than in conventional X-rays. However, Henckel et al. [39] reported that specific protocols that combine filters and image post-processing on multiple detector helical CT scans could reduce the radiation dose to a level comparable to standard radiographs. Nevertheless, the total cost per patient with THA may increase with 3D templating [40].

## Conclusion

The main advantage of 3D-based planning is higher accuracy, especially when dealing with obese patients. However, this process is associated with an extended planning time and increased exposure to radiation. Nonetheless, 3D templating is justifiable for patients with expected intra-operative difficulties caused by high BMI owing to its significantly higher accuracy compared with 2D templating.

## Data Availability

The datasets used and analysed during the current study are available from the corresponding author on reasonable request.
